# Inflammatory Fabry Cardiomyopathy Demonstrated Using Simultaneous [^18^F]-FDG PET-CMR

**DOI:** 10.1016/j.jaccas.2023.101863

**Published:** 2023-05-06

**Authors:** Christopher Orsborne, Jose M. Anton-Rodrigez, Neal Sherratt, Amy Watkins, Maelene Lohezic, David Clark, William Lloyd, Josephine H. Naish, Peter Woolfson, Anna B. Reid, Matthias Schmitt, Sivakumar Muthu, Parthiban Arumugam, Ana Jovanovic, Christopher A. Miller

**Affiliations:** a Division of Cardiovascular Sciences, School of Medical Sciences, Faculty of Biology, Medicine and Health, Manchester Academic Health Science Centre, University of Manchester, Manchester, United Kingdom; b Manchester University NHS Foundation Trust, Manchester, United Kingdom; c Salford Royal NHS Foundation Trust, Salford, United Kingdom; d GE Healthcare, Manchester, United Kingdom; e Division of Cell-Matrix Biology & Regenerative Medicine, Wellcome Centre for Cell-Matrix Research, School of Biology, Faculty of Biology, Medicine & Health, Manchester Academic Health Science Centre, University of Manchester, Manchester, United Kingdom

**Keywords:** cardiovascular magnetic resonance, Fabry disease, [^18^F]-fluorodeoxyglucose positron emission tomography, myocardial fibrosis, myocardial inflammation

## Abstract

Using hybridized [^18^F]-fluorodeoxyglucose positron emission tomography with cardiac magnetic resonance, we identify active myocardial inflammation and demonstrate its relationship with late gadolinium enhancement, in Fabry disease. We demonstrate that late gadolinium enhancement represents, at least in part, active myocardial inflammation and identify an early inflammatory phenotype that may represent a therapeutic window before irreversible tissue injury and adaptation occur. (**Level of Difficulty: Intermediate.**)

## Central Illustration

**Figure:**
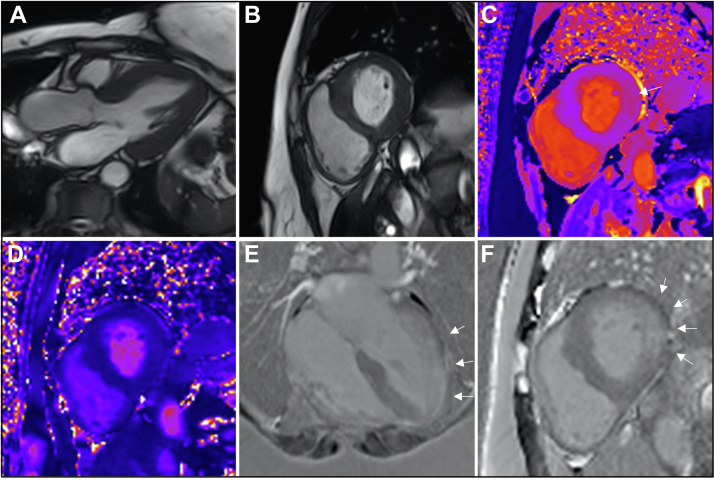

Fabry disease is a rare, X-linked, lysosomal storage disorder characterized by a deficiency of α-galactosidase and the accumulation of its substrate globotriaosylceramide (Gb3). Clinical cardiac manifestations are common, affecting both male and female patients, and they represent the leading cause of death.

**Learning Objectives**

• To recognize that LGE in Fabry disease is associated with myocardial collagen deposition and is thus used clinically to indicate myocardial fibrosis and observe that the origin of myocardial fibrosis in Fabry disease remains unclear.
• To recognize that hybridizing [^18^F]-FDG PET with CMR, and with CT, potentially provides unique insight into myocardial inflammation in the context of the other pathologic features.
• To observe that colocalization of myocardial [^18^F]-FDG uptake with LGE demonstrates that LGE in Fabry disease represents, at least in part, active myocardial inflammation and to recognize that myocardial inflammation may be reversible, thus representing a therapeutic window before irreversible tissue injury and adaptation occur.
• To observe that localization of myocardial [^18^F]-FDG uptake in the basal inferolateral wall in the absence of LGE may indicate an early Fabry phenotype characterized by myocardial inflammation, which may be a precursor to the development of myocardial fibrosis.
• To deduce that hybridized [^18^F]-FDG PET CMR provides unique insight into Fabry myocardial pathophysiology, which could be used to guide the development of new therapies and clinical guidelines, but considerable barriers exist to widespread clinical adoption.

Gb3 persistently activates multiple inflammatory and immunologic pathways, thus resulting in expression of inflammatory cytokines and cell adhesion molecules, oxidative stress, and apoptosis, and it is hypothesized to be central to disease progression.^1^

The pathologic findings of Fabry disease include myocardial hypertrophy and fibrosis. The latter, which may be associated with adverse outcomes such as ventricular arrhythmia and sudden cardiac death, can be identified using cardiovascular magnetic resonance (CMR) late gadolinium enhancement (LGE), characteristically involving the left ventricular basal inferolateral wall.^2^ The origin of myocardial fibrosis in Fabry disease remains unclear.

Hybridizing [^18^F]-fluorodeoxyglucose ([^18^F]-FDG) positron emission tomography (PET) with CMR, and with computed tomography (CT), potentially provides unique insight into myocardial inflammation in the context of the other pathologic features.

Using dual hybridized [^18^F]-FDG PET-CMR and [^18^F]-FDG PET-CT, we identified active myocardial inflammation, and demonstrated its relationship with LGE, in 2 patients with Fabry disease. This work was approved by an ethics committee. Participants provided written informed consent (NCT03949920).

## Case Descriptions

### Case 1

**Figure 1:**
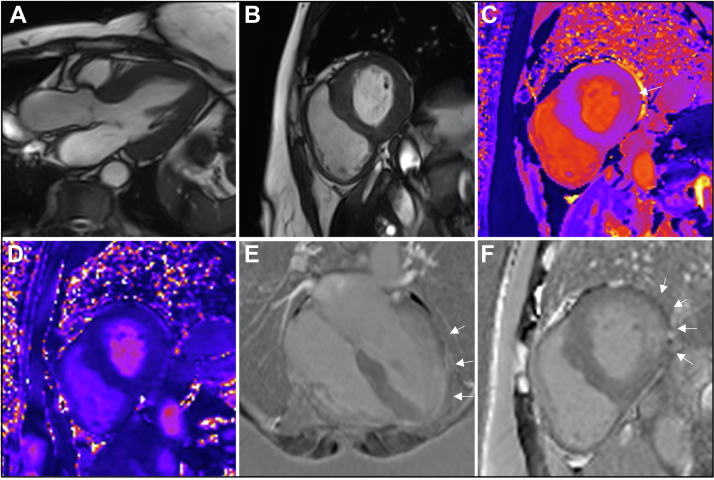
Case #1 Cardiac Magnetic Resonance Images **(A)** Three-chamber and **(B)** basal short-axis balanced steady-state free precession images. **(C)** Basal short-axis T_1_ map (modified look-locker inversion recovery). The **arrow** indicates an area of high native myocardial T_1_ relaxation time. **(D)** Basal short-axis T_2_ map. **(E)** Four-chamber phase-sensitive inversion recovery late gadolinium enhancement image acquired in the late phase following gadolinium administration. **(F)** Basal short-axis phase-sensitive inversion recovery late gadolinium enhancement image. The **arrows** indicate areas of late gadolinium enhancement. The basal short-axis T_1_ map, the basal short-axis T_2_ map, and the basal short-axis phase-sensitive inversion recovery late gadolinium enhancement images were acquired in identical imaging planes. Images are from a cardiac magnetic resonance scan conducted before the hybridized imaging.

A 68-year-old woman with no relevant past medical history was found to have Fabry disease (GLA mutation: c.641C>T, p.Pro214Leu; α-galactosidase levels, 3.0 μmol/L/h [normal 1.2-50 μmol/L/h]) following a diagnosis in a family member. CMR confirmed left ventricular hypertrophy (Figures 1A and 1B, Videos 1 and 2) and demonstrated dense, nonischemic LGE in the basal inferolateral wall and in the midapical and apical anterolateral wall (Figures 1E and 1F). T_1_ and T_2_ relaxation times were normal, although T_2_ was higher in regions with LGE compared with the septum (40 ms vs 44 ms) (Figures 1C and 1D). Further CMR measurements are detailed in Table 1.^1^

**Table 1:** Summary of Clinical and Imaging Parameters From the Cases Presented

	Patient 1	Patient 2
LVEDVI, mL/m^2^	67.0	106.2
LVESVI, mL/m^2^	15.5	48.2
LVMWT, mm	17.7	16.2
LVMI, g/m^2^	77.9	91.2
Global longitudinal strain, %	−12.7	−12.4
LVEF, %	77	55
RVEF, %	66	70
Native myocardial T_1_,^a^ ms	1,154	1,216
Myocardial T_2_,^b^ ms	41	41
Late gadolinium enhancement^c^		
Infarct, n, %	0	0
Atypical (nonischemic), n, %	12.3	0.9^d^
Atypical LGE percentage, %	9.2	0.4^d^
ECV, %	27.3	24.1

ECV = extracellular volume fraction (measured using a same day hematocrit); LVEDVI = left ventricular end-diastolic volume indexed; LVEF = left ventricular ejection fraction; LVESVI = left ventricular end-systolic volume indexed; LVMI = left ventricular mass index (including papillary muscle mass); LVMWT = left ventricular maximal wall thickness; RVEF = right ventricular ejection fraction.

a Myocardial T_1_ was measured from the ventricular septum in basal and midventricular short-axis T_1_ maps.

b T_2_ relaxation time was measured from the middle third of myocardium in basal and midventricular short-axis T_2_ maps.^1^

c The presence of LGE was agreed on by 2 experienced operators; if LGE was present, LGE mass was quantified using a signal intensity threshold of >5 SDs above an area of remote myocardium.^1^

d Nonspecific right ventricular insertion point fibrosis only. Measurements are from a cardiac magnetic resonance scan conducted before the hybridized imaging (3.0-T Skyra scanner, Siemens Medical Imaging).

**Figure 2:**
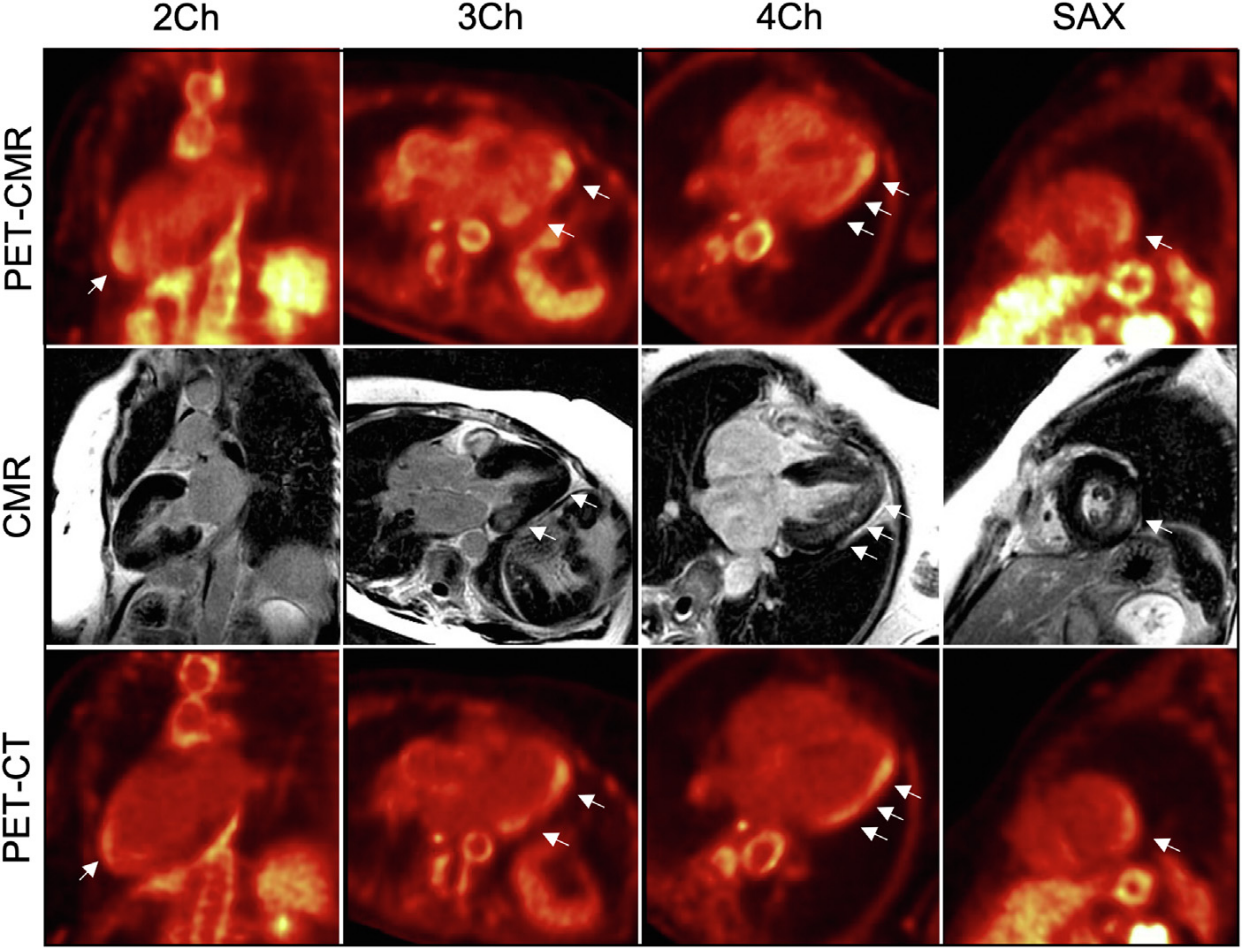
Case #1 Hybridized Images **(Top row)** [^18^F]-fluorodeoxyglucose ([^18^F]-FDG) positron emission tomography (PET) with cardiac magnetic resonance (CMR). **(Middle row)** Cardiac magnetic resonance. **(Bottom row)** [^18^F]-FDG positron emission tomography with computed tomography (CT). On the [^18^F]-FDG positron emission tomography with cardiac magnetic resonance and [^18^F]-FDG positron emission tomography with computed tomography images, the **arrows** indicate areas of increased [^18^F]-FDG uptake. On the cardiac magnetic resonance images, the **arrows** indicate areas of late gadolinium enhancement. Individual modality images rather than hybridized images are presented throughout to facilitate visualization of the abnormalities identified with each modality. 2Ch = 2-chamber view; 3Ch = 3-chamber view; 4Ch = 4-chamber view; SAX = short-axis view.

[^18^F]-FDG PET-CMR showed focal [^18^F]-FDG uptake, which colocalized with LGE in the basal inferolateral wall and the midapical anterolateral wall, but which was also present in the apical anterior wall, apical cap, and apical inferolateral wall, where LGE was absent (Figure 2, top and middle rows). An [^18^F]-FDG PET-CT scan confirmed the extent and distribution of [^18^F]-FDG uptake (Figure 2, bottom row). Migalastat therapy was commenced, and the patient was assessed in clinic after 12 months and remained clinically well.

### Case 2

**Figure 3:**
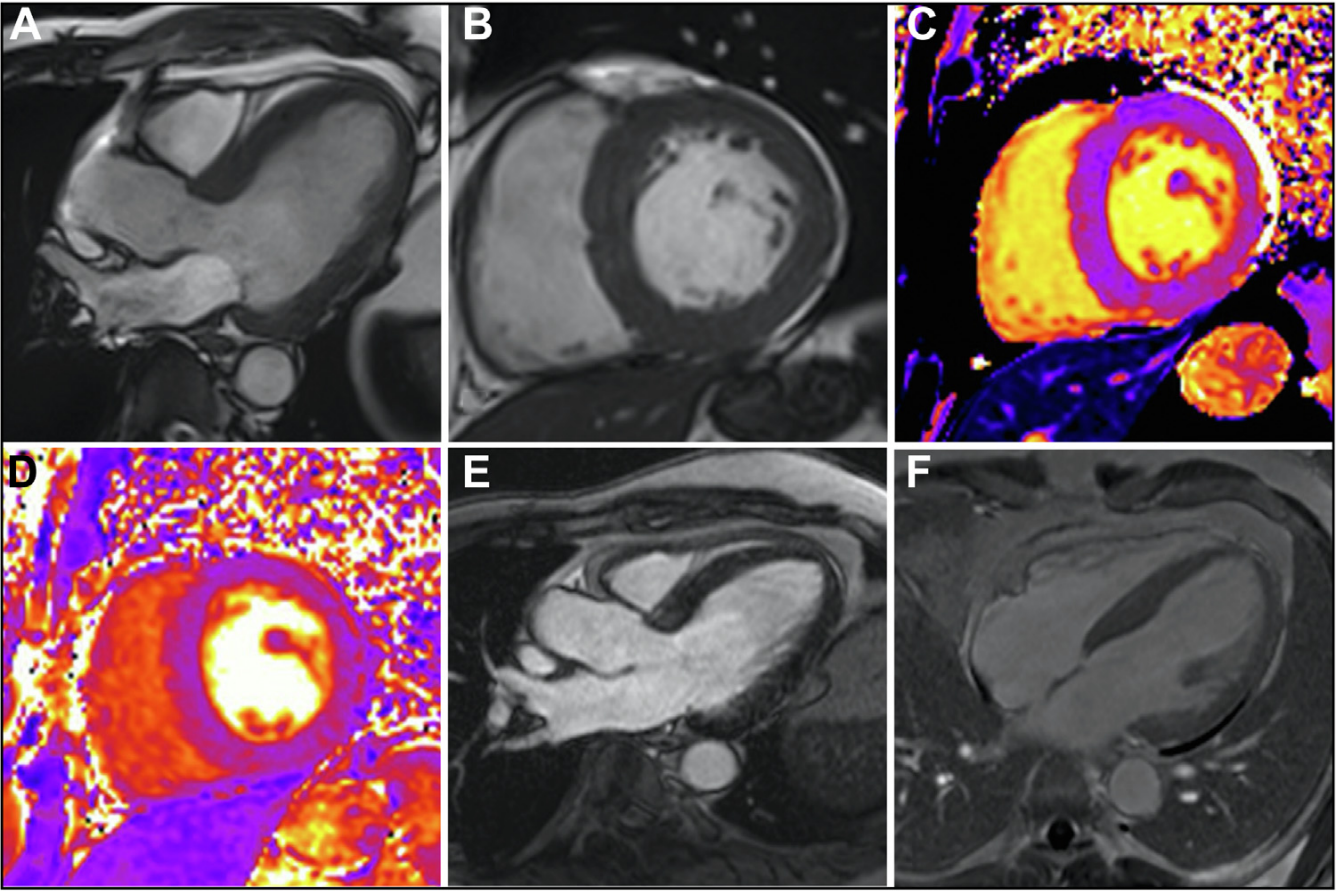
Case #2 Cardiac Magnetic Resonance Images **(A)** Three-chamber and **(B)** basal short-axis balanced steady-state free precession images. **(C)** Basal short-axis T_1_ map (modified look-locker inversion recovery). **(D)** Basal short-axis T_2_ map. **(E)** Three-chamber phase-sensitive inversion recovery late gadolinium enhancement image acquired in the late phase following gadolinium administration. **(F)** Basal short-axis phase-sensitive inversion recovery late gadolinium enhancement image. Images are from a cardiac magnetic resonance scan conducted before the hybridized imaging.

A 54-year-old man with a history of medicated hypertension and recurrent, excised melanoma was found to have Fabry disease following an incidental finding of renal impairment with proteinuria and subsequent renal biopsy (GLA mutation: c. 1066C>T, p.Arg356Trp; α-galactosidase levels, 4.4 nmol/L/h [normal 4-21.9 nmol/L/h]). CMR confirmed left ventricular hypertrophy (Figures 3A and 3B, Videos 3 and 4) but demonstrated no LGE (other than minor right ventricular insertion point fibrosis, a nonspecific finding) (Figures 3E and 3F). T_1_ and T_2_ relaxation times were normal (Figures 3C and 3D). Further CMR measurements are detailed in Table 1.

**Figure 4:**
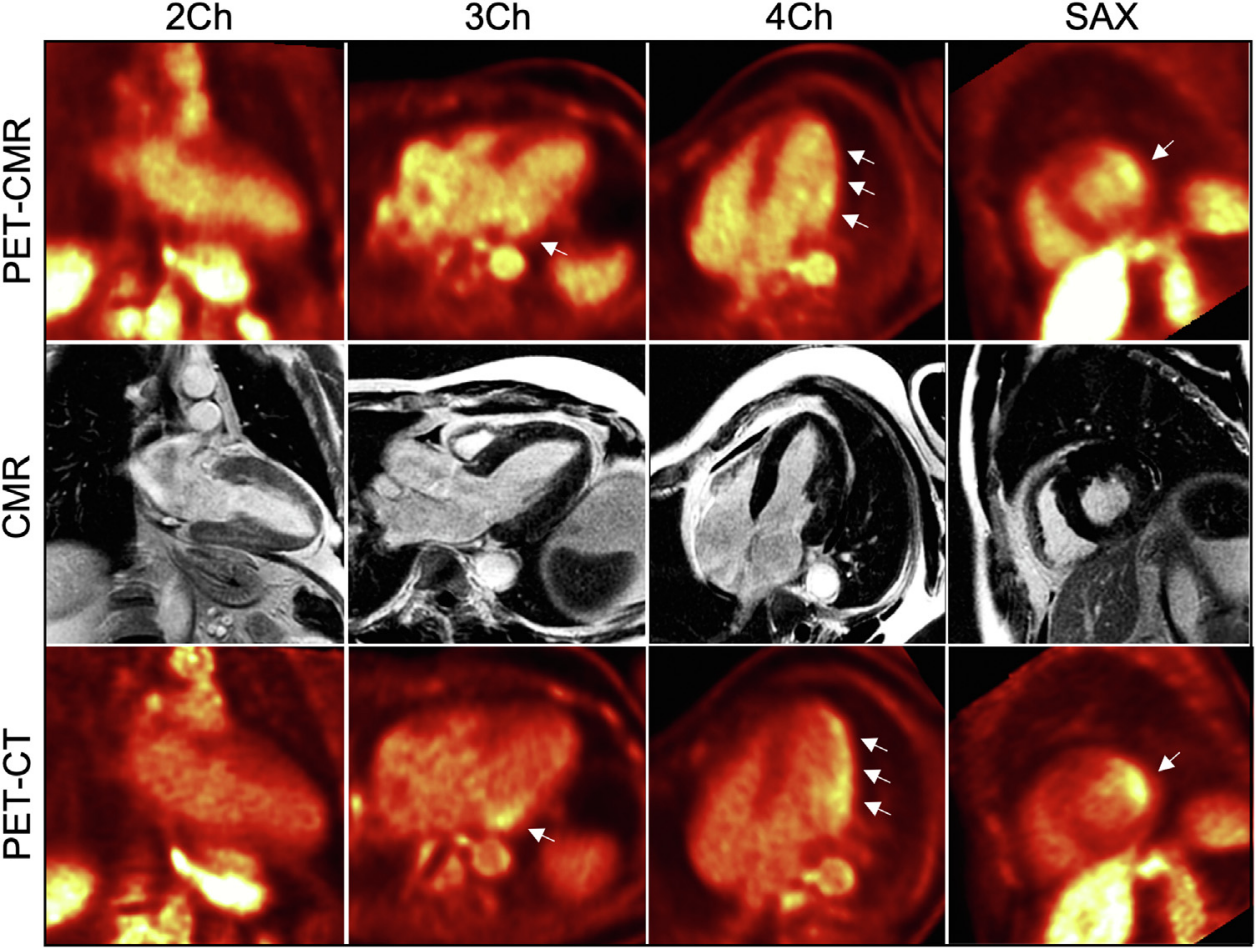
Case #2 Hybridized Images **(Top row)** [^18^F]-fluorodeoxyglucose ([^18^F]-FDG) positron emission tomography (PET) with cardiac magnetic resonance (CMR). **(Middle row)** Cardiac magnetic resonance. **(Bottom row)** [^18^F]-FDG positron emission tomography with computed tomography (CT). On the [^18^F]-FDG positron emission tomography with cardiac magnetic resonance and [^18^F]-FDG positron emission tomography with computed tomography images, the **arrows** indicate areas of increased [^18^F]-FDG uptake. Individual modality images rather than hybridized images are presented throughout to facilitate visualization of the abnormalities identified with each modality. Abbreviations as in Figure 2.

**Figure 5:**
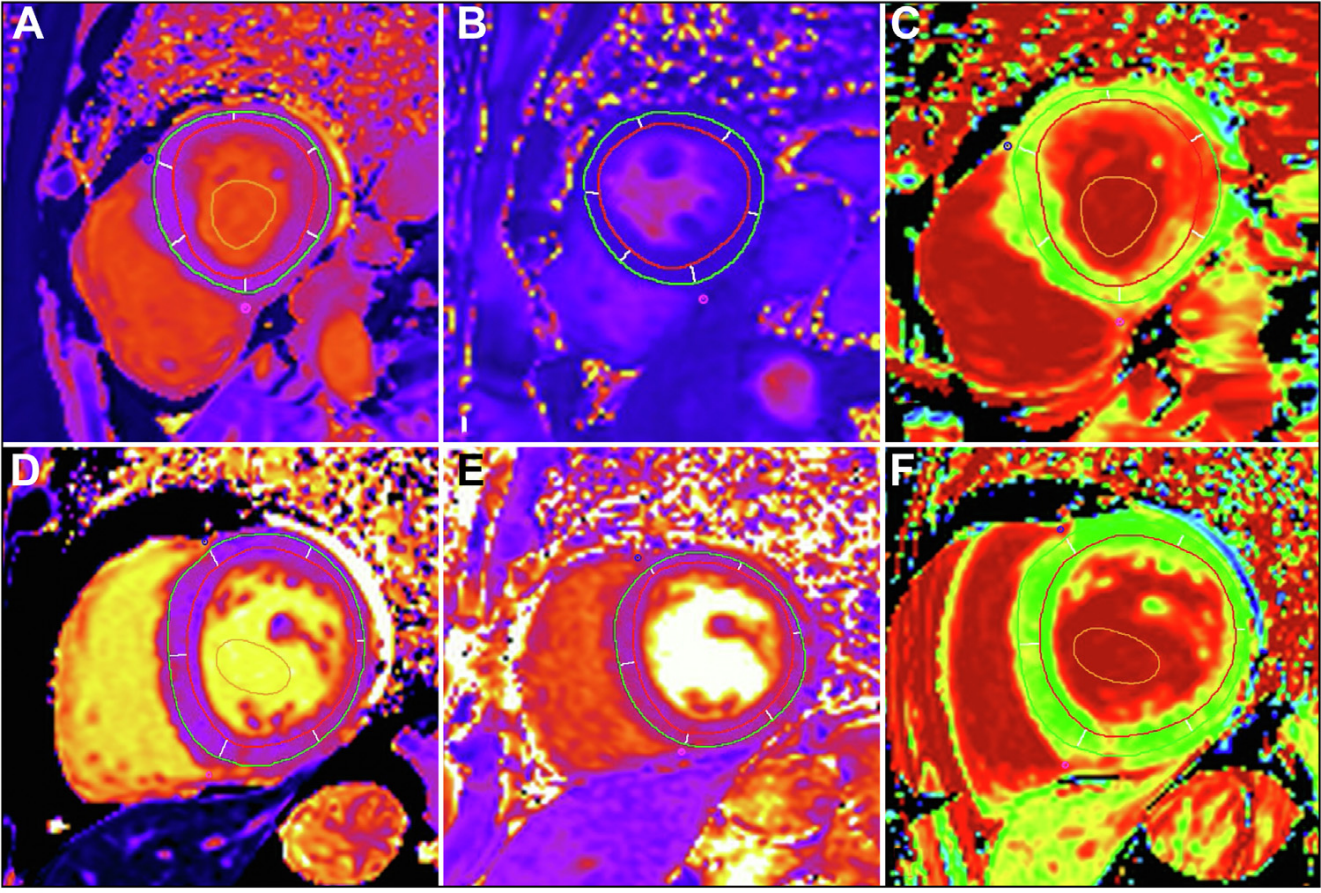
Native T_1_, T_2_, and Extracellular Volume Maps **(Top row)** Case #1. **(Bottom row)** Case #2. **(A and D)** are native T_1_ maps, **(B and E)** are T_2_ maps, and **(C and F)** are extracellular volume maps acquired from the basal short-axis slice. Regions of interest are annotated on the relevant maps. Native myocardial T_1_ relaxation time, myocardial T_2_ relaxation time, and extracellular volume fraction within the [^18^F]-fluorodeoxyglucose–enhancing segments were as follows: case #1: 1,350.4 ms, 45.9 ms, and 42.1%, respectively, within the inferolateral segment; case #2, 1,224.9 ms, 40.1 ms, and 23.2%, respectively, within the anterolateral segment and 1,255.0 ms, 41.5 ms, and 24.9%, respectively, within the inferolateral segment.

**Figure 6:**
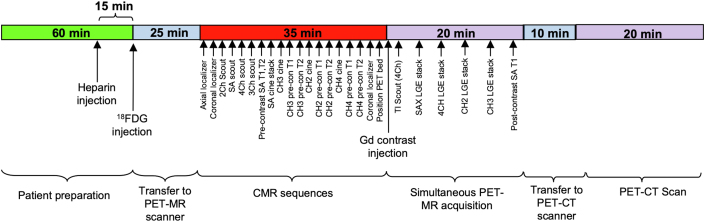
Schematic of the Simultaneous Hybridized [^18^F]-FDG-PET-CMR Workflow CH2 = 2-chamber; CH3 = 3-chamber; CH4 = 4-chamber; Gd = gadolinium; LGE = late gadolinium enhancement; MR = magnetic resonance; pre-con = precontrast; SA = short-axis; other abbreviations as in Figure 2.

PET-CMR showed focal [^18^F]-FDG uptake in the lateral wall in the absence of LGE (Figure 4, top and middle rows). [^18^F]-FDG PET-CT confirmed the extent and distribution of the [^18^F]-FDG uptake (Figure 4, bottom row). Left ventricular ejection fraction was mildly reduced. Impaired left ventricular ejection fraction is a feature of Fabry cardiomyopathy, which could possibly be related to chronic inflammation, but that is unknown. Migalastat treatment was commenced but was subsequently switched to enzyme replacement therapy because of a decline in renal function. Tissue mapping regions of interest are demonstrated in Figure 5. A schematic of the simultaneous hybridized [^18^F]-FDG PET-CMR workflow is presented in Figure 6. Patients underwent 24 hours of a low-carbohydrate diet and a 6-hour fast before [^18^F]-FDG administration.

## Discussion

Gb3 persistently activates multiple inflammatory and immunologic pathways, thereby resulting in increased expression of inflammatory cytokines and cell adhesion molecules, oxidative stress, and apoptosis, and it is hypothesized to be central to Fabry disease progression.^2^

The presented cases demonstrate 2 key findings:

First is colocalization of myocardial [^18^F]-FDG uptake with LGE. LGE represents localized relative expansion of the extracellular space, of which there are multiple causes, including inflammation and fibrosis. In the classic histologic validation by Moon et al,^3^ LGE in Fabry disease is associated with myocardial collagen deposition, and LGE is thus used clinically to indicate myocardial fibrosis.

However, in keeping with the findings of Nappi et al,^4^ and Spinelli et al,^5^ the colocalization of myocardial [^18^F]-FDG uptake with LGE demonstrates that LGE in Fabry disease represents, at least in part, active myocardial inflammation. This finding is important because active myocardial inflammation may be reversible, thus representing a therapeutic window before irreversible tissue injury and adaptation occur.

Current disease-modifying therapies do not specifically target inflammation. Furthermore, current consensus generally advocates LGE as an indication for initiating disease-modifying therapy but advises against therapy initiation in the presence of extensive LGE because it is generally thought to reflect irreversible myocardial fibrosis. The current cases demonstrate that, at least in some patients, this may not be true.

The second key finding is myocardial [^18^F]-FDG uptake in the basal inferolateral wall in the absence of LGE. These intriguing findings are hypothesized to represent an early Gb3-mediated myocardial inflammation phenotype, which may be a precursor to the development of myocardial fibrosis. The absence of pathologic LGE and also normal T_2_ suggest that myocardial edema may not be a feature of Fabry myocardial inflammation, at least at this stage. This hypothesis is supported by a previous study that demonstrated elevated T_1_ times in the lateral wall in patients with focal [^18^F]-FDG uptake, compared with patients without focal [^18^F]-FDG uptake, even in the absence of LGE and elevated T_2_.^6^ It will be interesting to observe whether the patient in case #2 goes on to have LGE in this region of myocardium.

Our cases also serve to demonstrate that [^18^F]-FDG PET-CMR provides unique insight into Fabry myocardial pathophysiology, which could be used to guide the development of new therapies and clinical guidelines. However, [^18^F]-FDG PET-CMR is cost, time, and resource intensive. It requires administration of radiation, a pertinent factor given the age range and particularly if repeat examinations are to be considered. It also requires strict adherence to a low-carbohydrate diet. CMR-based attenuation correction is challenging, hence the corroboration with contemporaneous PET-CT, but this adds to the radiation dose. Coregistration of CMR and PET-CT images may be more practical, and more cost-effective, in patients already assessed with CMR. Nevertheless, [^18^F]-FDG PET-CMR represents an attractive “1-stop shop.”

## Conclusions

Using dual hybridized [^18^F]-FDG PET CMR and [^18^F]-FDG PET CT, we identified active myocardial inflammation and demonstrated its relationship with LGE in 2 patients with Fabry disease. The presented cases demonstrate that LGE represents, at least in part, active myocardial inflammation and also reveals inflammation in the absence of LGE, which suggests an early Gb3-mediated myocardial inflammation phenotype as a precursor to myocardial fibrosis. Active myocardial inflammation may be reversible, thus representing a therapeutic window before irreversible tissue injury and adaptation occur.

## Funding Support and Author Disclosures

This work is part of a study funded by Amicus Therapeutics. Amicus had no role in the design and conduct of the study; collection, management, analysis, and interpretation of the data; preparation or approval of the manuscript; or the decision to submit the manuscript for publication. This work was also supported in part by a British Heart Foundation Accelerator Award to the University of Manchester (AA/18/4/34221). Dr Orsborne has received research support from Amicus Therapeutics. Dr Lohezic has been formerly employed by GE Healthcare; and has served as a contractor for GE Healthcare. Dr Schmitt has received research support from Amicus Therapeutics. Dr Jovanovic has received research support from Amicus Therapeutics. Dr Miller has received funding through an Advanced Fellowship from the National Institute for Health Research (NIHR; NIHR301338) (The views expressed in this publication are those of the authors and not necessarily those of the NIHR, the National Health Service, or the UK Department of Health and Social Care); acknowledges support from the University of Manchester British Heart Foundation Accelerator Award (AA/18/4/34221) and the NIHR Manchester Biomedical Research Centre (NIHR203308); has participated on advisory boards/consulted for AstraZeneca, Boehringer Ingelheim and Lilly Alliance, Novartis and PureTech Health; has served as an advisor for HAYA Therapeutics; has received speaker fees from AstraZeneca, Boehringer Ingelheim and Novo Nordisk; has received conference attendance support from AstraZeneca and has received research support from Amicus Therapeutics, AstraZeneca, Guerbet Laboratories Limited, Roche and Univar Solutions B.V. All other authors have reported that they have no relationships relevant to the contents of this paper to disclose.

## Appendix

Video 1 Balanced Steady-State Free Precession cine acquired in the 3-Chamber View Derived From Case #1

Video 2 Balanced Steady-State Free Precession Cine Acquired in the Short-Axis View From Base to Apex Derived From Case #1

Video 3 Balanced Steady-State Free Precession Cine Acquired in the 3-Chamber View Derived From Case #2

Video 4 Balanced Steady-State Free Precession Cine Acquired in the Short-Axis View Derived From Case #2
